# Flexibility of Continental Navigation and Migration in European Mallards

**DOI:** 10.1371/journal.pone.0072629

**Published:** 2013-08-30

**Authors:** Mariëlle L. van Toor, Anders Hedenström, Jonas Waldenström, Wolfgang Fiedler, Richard A. Holland, Kasper Thorup, Martin Wikelski

**Affiliations:** 1 Department of Migration and Immuno-Ecology, Max Planck Institute for Ornithology,Radolfzell, Germany; 2 Department of Biology, University of Konstanz, Konstanz, Germany; 3 Department of Biology, Lund University, Lund, Sweden; 4 Centre for Ecology and Evolution in Microbial Model Systems (EEMiS), Linnaeus University, Kalmar, Sweden; 5 School of Biological Sciences, Queen’s University Belfast, United Kingdom; 6 Center for Macroecology, Evolution and Climate, Natural History Museum of Denmark, University of Copenhagen, Copenhagen, Denmark; CNRS, Université de Bourgogne, France

## Abstract

The ontogeny of continent-wide navigation mechanisms of the individual organism, despite being crucial for the understanding of animal movement and migration, is still poorly understood. Several previous studies, mainly conducted on passerines, indicate that inexperienced, juvenile birds may not generally correct for displacement during fall migration. Waterbirds such as the mallard (*Anas platyrhynchos*, Linnaeus 1758) are more flexible in their migration behavior than most migratory songbirds, but previous experiments with waterbirds have not yet allowed clear conclusions about their navigation abilities. Here we tested whether immature mallard ducks correct for latitudinal displacement during fall migration within Europe. During two consecutive fall migration periods, we caught immature females on a stopover site in southeast Sweden, and translocated a group of them ca. 1,000 km to southern Germany. We followed the movements of the ducks via satellite GPS-tracking and observed their migration decisions during the fall and consecutive spring migration. The control animals released in Ottenby behaved as expected from banding recoveries: they continued migration during the winter and in spring returned to the population’s breeding grounds in the Baltics and Northwest Russia. Contrary to the control animals, the translocated mallards did not continue migration and stayed at Lake Constance. In spring, three types of movement tactics could be observed: 61.5% of the ducks (16 of 26) stayed around Lake Constance, 27% (7 of 26) migrated in a northerly direction towards Sweden and 11.5% of the individuals (3 of 26) headed east for ca. 1,000 km and then north. We suggest that young female mallards flexibly adjust their migration tactics and develop a navigational map that allows them to return to their natal breeding area.

## Introduction

The integration of knowledge about navigation, migration routes and the environmental factors triggering migration decisions are essential to ultimately understand by which means birds decide if, when, and where to migrate. Environmental changes and the resulting shifts in the timing of life history stages [1] make the understanding of individual movement decisions more important than ever. Changes in migratory movement can be rapid and drastic, such as the shift of wintering areas seen in blackcaps (*Sylvia atricapilla*, Linnaeus 1758) in Europe over the last 50 years [2], or the suggested decrease of synchrony between Common cuckoos (*Cuculus canorus*, Linnaeus 1758) and their short-distance migratory host species [3], [4]. Furthermore, it is thought that a continuous warming of winters might drastically decrease the proportion of migrants not only in partially migratory species [5], [6], but also in obligate migrants, though exceptions do exist [7]. The principles of bird navigation have been studied for decades, and although significant progress has been made, we still do not fully understand the mechanisms involved and their integration into the system [8], [9]. Experiences made during early lifestage might also influence later decisions, thus it is crucial to know the ontogeny of individuals to understand it’s behaviour later in life. This might also be relevant for navigation and migration. Thus, an intensive part of the navigation research field has been the study on how juvenile birds find their way to their wintering grounds during their first migration [10]–[12]. Guidance by parents and experienced conspecifics can explain how inexperienced birds can find their species-specific wintering grounds in social migrants like storks or geese [13]–[15]. However, many migratory bird species are non-social migrants, thus, other mechanisms must be involved. It was in the 1950s when the theory of vector or clock-and-compass navigation found its first support in a field study involving the translocation of thousands of starlings [10]. The experiment has been repeated in a variety of species, mostly songbirds, and although most of them found evidence supporting the principle of vector navigation [16], [17], some studies found contradicting results [12], [18] Thus, we cannot generalize the concept of vector navigation over species.

The factors triggering the decision if and when to migrate are also not clear. Several potential factors have been identified for spring arrival of songbirds on their breeding grounds in Europe [19], such as local weather, large scale weather patterns and vegetation greenness. Adverse weather conditions, snow cover and depletion of food resources can induce migration in facultative migrants [20]–[22] Similarly, general winter harshness has been shown to influence the tendency of ducks to migrate in winter [23]–[25], a behavior that might be induced by the freezing of lakes.

**Figure 1:**
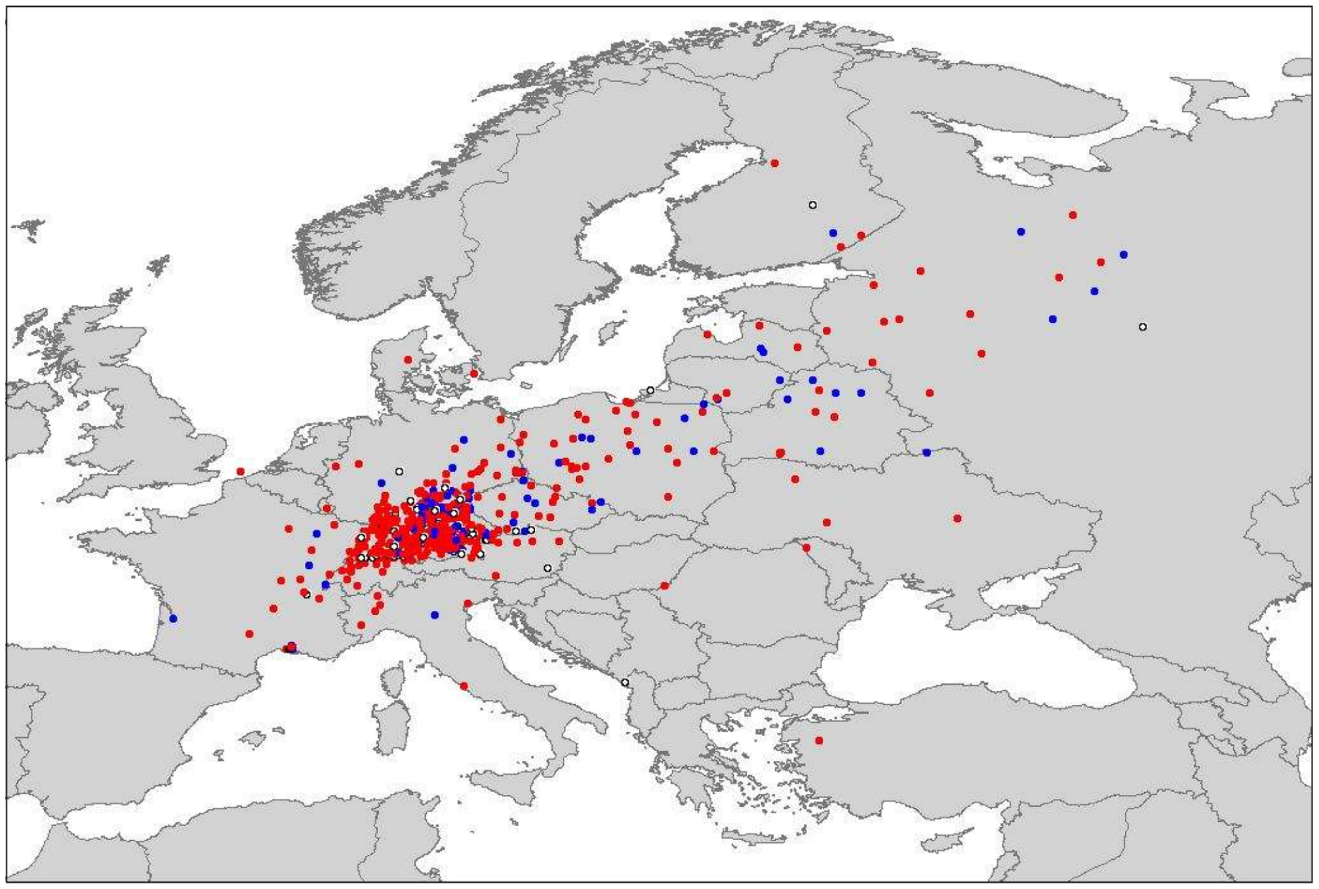
Recovery sites of banded mallards that were either ringed in Southern Germany or ringed abroad and recovered in Southern Germany. Blue points are females, red points represent males. The banding data was collected by the ringing center Radolfzell, Germany, from 1947–2011.

This information is crucial in the light of emerging infectious diseases, as ducks, and waterbirds in general, are thought to be the main reservoirs for avian influenza A virus (AIV). Especially mallards (*Anas platyrhynchos*, Linnaeus 1758) have the potential to transport the virus over long distances, as they can shed considerable amounts of highly pathogenic AIV while remaining asymptomatic [26]. Thus knowledge about mallard movements, especially winter movements and how they are induced, could benefit the general understanding of the population dynamics with respect to migration, and although the mallard is a comprehensively studied species, little is known about individual migratory decisions. Mallards are more likely to migrate in cold than in mild winters [23], [24], and also migrate further south under harsher conditions [27], indicating that this species can flexibly adjust to local conditions. This is also exemplified by the recovery of banded individuals from Germany (see figure 1), showing that although northeast-southwest migration corridor is visible, there is a lot of variation. Similar findings have been revealed by molecular data [28].

The aim of this study was to bring together information about navigation and migration decisions using a setup including a translocation experiment, the use of environmental data, and GPS-tracking of individuals. We wanted to use a species that adjusts its migration to weather conditions, and for which social influence on migration is less pronounced than in typical social migrants, for example storks. Mallards in Northern Europe are usually migratory [29], and respond to local environmental conditions [23], [24], [27]. Thus, juvenile mallards are suitable study subjects. Because an important social influence on migration has been shown for mallard drakes [30], we chose only females. Prior to this experiment, we had a number of expectations. Using mallards from the usually migratory population of Northern Europe, we expected to see migratory activity also after translocation, especially after temperature drops. Furthermore, we expected the migratory behavior in the spring after the translocation and release to be unaffected by the previous displacement. Thus the ducks should migrate back to their breeding grounds, similar to what European Starlings did in previous experiments [10]. Overall, the main aim was to get an overview of the navigational skills of a partially migratory and flexible species, its tendency to migrate at given environmental conditions and the factors that may be affecting the decision to migrate, or not.

## Results

### Migratory Behavior and Initial Migration Directions

**Figure 2:**
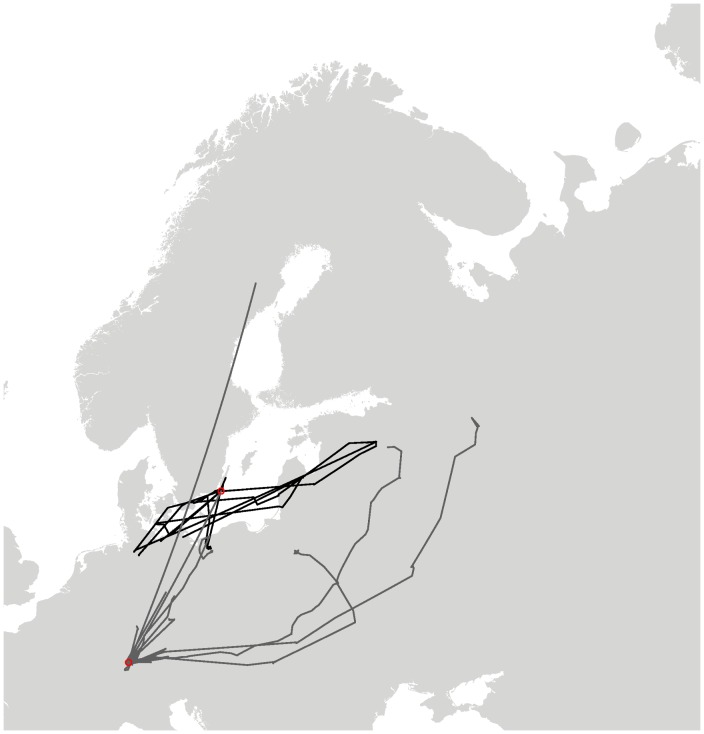
Map of all tracked individuals. Black lines indicate animals released in Ottenby (n = 14), grey lines represent animals translocated to and released at the Lake of Constance (n = 26). The release locations are highlighted by red circles.

**Figure 3:**
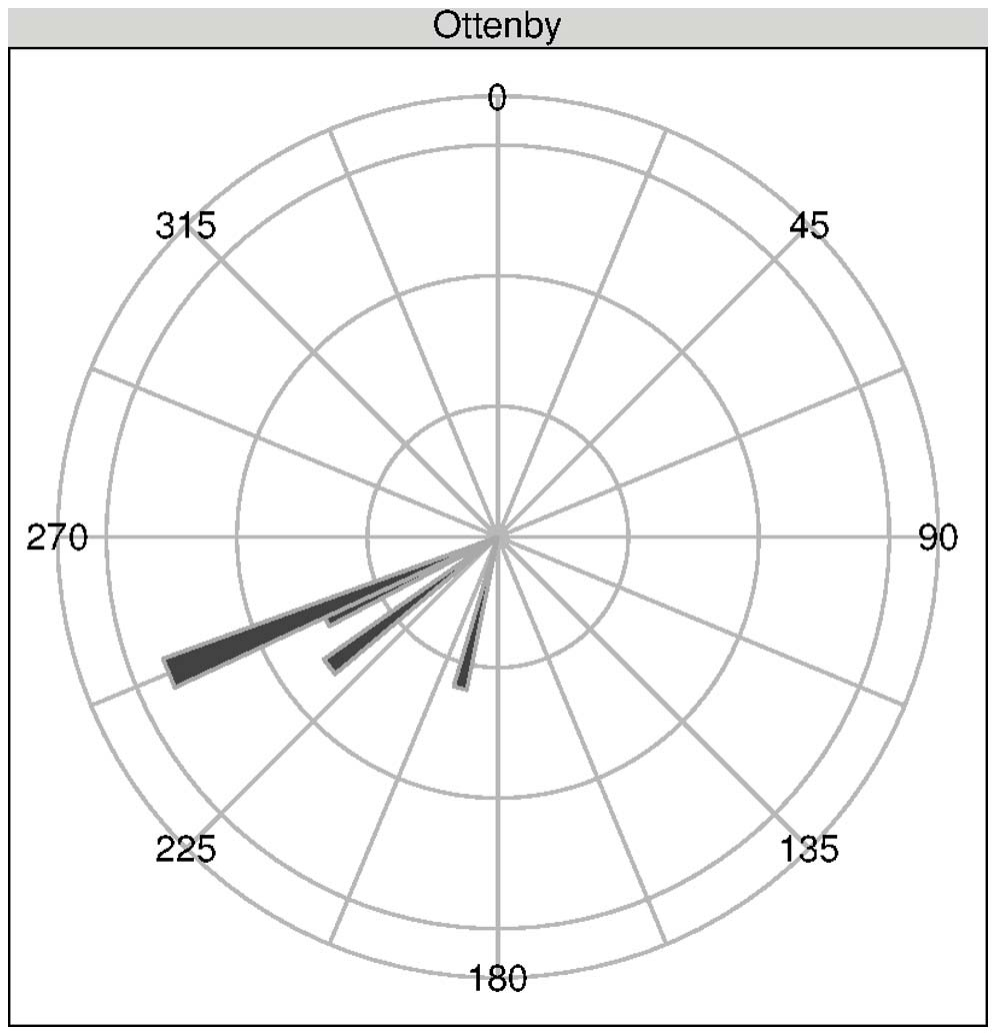
Mean heading of winter migration for control birds in Ottenby (n = 4). The distance traveled to the final wintering sites in indicated by the length of the bars.

**Figure 4:**
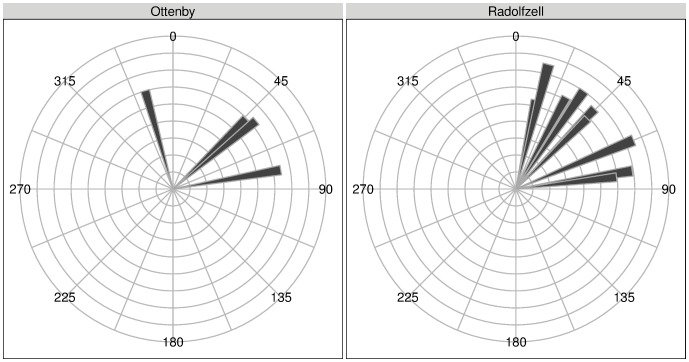
Initial headings for spring migrations for both animals released in Ottenby (left panel, n = 4) and Radolfzell (right panel, n = 10). The log(distance) traveled in this initial direction is represented by the length of the bars.

Eighteen individual migration events could be identified (see Figure 2), of which four were classified as winter migration (Ottenby: 4, Radolfzell: 0), and the remaining 14 as spring migratory events (Ottenby: 4, Radolfzell: 10). The most obvious finding was that none of the ducks released in Radolfzell continued migration, whereas four of the control birds did (Fisher’s exact test, 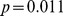). Therefore, no comparison between migration directions was possible for winter. The observed migrations for Ottenby were in line with the expectations from banding recoveries of birds from the area [31]. This also indicates that the tags did not qualitatively influence the bird’s normal migration behavior. One of the migratory animals, however, did not head southwest to Denmark and northern Germany, but straight south, and wintered in northern Poland, which has also been observed before by banding efforts [31]. The mean migration directions of the birds during winter migration are shown in Figure 3. Although we did not observe the continuation of south- or south-westward migration in Radolfzell, smaller movements of the translocated ducks could be observed. The part of the lake the ducks were released into is very food-rich, but also shallow. Especially during the first study winter, large parts of the lake surface were covered with ice for several weeks in January and February (personal observation). The freezing of the shallow areas coincided with the ducks moving to deeper parts of Lake Constance, and the mouths of surrounding streams. On another occasion, one single duck left the surroundings of the area and traveled as far as to a Swiss lake about 40 km away, but returned to Lake Constance the following day. During spring, the proportion of migratory birds did not differ between sites (Fisher’s exact test, 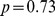). We only saw uniformity of the directions for the initial mean heading of control ducks in spring (Rayleigh’s test of uniformity, 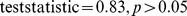), for all other subsets uniformity could not be verified (Rayleigh’s test of uniformity, all 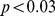). This means that differing spring migration headings both within treatment groups, and between treatment groups, were observable. Mean migration directions, both total and initial, for both seasons and groups are listed in Table 1. Initial headings for both groups are depicted in Figure 4. Due to the low sampling rate during winter, the initial headings of Ottenby birds during winter migration are identical to the final headings, and are thus being ignored. This difference between initial and final heading for the Radolfzell birds seem to be driven by mainly three animals, who, compared to most other migratory birds, first headed east and turned more northerly after having traveled several hundred km. All end locations of the tracks are within the expected area to find female mallards banded in Southern Germany and recovered during spring (see Figure 1). Another interesting finding was the recovery of one duck in northern Sweden after it was being shot in August 2009, where usually no birds from either Ottenby [31] or Southern Germany (see Figure 1) are found. The satellite tag had long stopped gaining positions by then (last GPS-fix 2008-12-09). Regarding the weather conditions, we found that temperatures were significantly lower in Radolfzell than in Ottenby during the complete period from December through April (one sample t-tests; 2008/09: 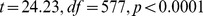; 2009/10: 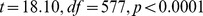). The mean difference in weekly mean temperatures between sites was 4.51°C in 2008/09 and 3.98°C in 2009/10. Similarly, the daily variation in temperatures was higher in Radolfzell than in Ottenby. Temperatures in Radolfzell were constantly lower until mid-/end-March in both years. This was also the period of rapid temperature increases, as well as the beginning of spring migration of the animals at both sites.

**Table 1:** Migration headings.

Site	total winter heading	initial spring heading	total spring heading
Ottenby	230.12±21.45	41.89±35.38	53.33±22.89
Radolfzell	–	44.37±16.44	39.64±24.24

Initial and total headings for both winter and spring migration. For winter migration, only total headings are given, as often not enough data points were available to distinguish an initial heading. Headings are given as mean and standard deviation.

## Discussion

Overall, only a small proportion of the ducks showed migratory activity, especially during winter. Although some migrations might not have been detected due to transmitter failure, this was a surprising finding. As we had expected similar responses of the animals to the general temperature conditions they experienced, it seems that temperature, or a drop in temperature alone is not sufficient to induce migratory movement in control ducks, although we could not statistically test this observation. This is supported by the finding that although absolute temperatures were significantly lower in Radolfzell than at Ottenby during the complete winter, none of the transported ducks migrated. Not even freezing of the shallow and food-rich stretches of Lake Constance was sufficient to induce migration to a level that was seen at Ottenby. We hypothesize that temperature alone does not have a great influence on the decision to migrate or not in mallards. We hypothesize that a combination of temperature and food availability/accessibility, and potentially also body condition are more likely to be the factors that ultimately lead to the decision. However, food availability is hard to estimate for an omnivorous species like the mallard, as it does depend on a variety of factors, like temperature and snow, and might vary drastically on small scales. Moreover, social factors might play a role, as both release sites are wintering sites used by large numbers of waterbirds. It should be noted, however, that the individuals under study were translocated forward along their usual migration direction. Forward translocation has repeatedly been shown to induce changes in behavior [32], [33]. Though we have no means to estimate the effects of this forward translocation on the migration decisions of individuals, we think that the reluctance of the test ducks to migrate is more a result of the suitability of local conditions at Lake Constance. We base this hypothesis on banding data and atble isotope analyses of mallards which show that mallards wintering and stopping over near Ottenby usually do not migrate to Lake Constance [31], [34]. In spring, migration patterns were more similar between sites, as the same proportions of birds in Ottenby and Radolfzell migrated approximately at the same time. The control birds migrated through the Ottenby area (from Southwest) and headed towards their presumed natural breeding grounds as expected [31]. Overall headings from the translocated birds did differ from the control ducks, and it seems as if two strategies for spring migration were detectable in the translocated ducks. Most ducks headed straight north-north-east, as if heading towards Ottenby, with one duck going as far as northern Sweden. Three of the transported ducks, however, first headed in a more easterly direction and turned northwards when reaching the longitudes of the area the control birds migrated to. It is unclear how these birds decided when to turn north, but the movement trajectories could be interpreted as if individuals had noticed that they were in the wrong place and then corrected for the southward translocation. Based on the observation that this second group of transported ducks ended up in their potential natural breeding grounds, and the first group had a more northerly heading than the control group, we conclude that mallards, just like the starlings from Perdeck’s original experiment [10], can correct for translocation during the spring season following the experiment. However, explanations other than true navigation might also be possible. As the spring migration routes of the translocated ducks are in line with expectations from birds banded in Southern Germany (see Figure 1), we cannot exclude social influences. Additionally, the true origin of the birds remains unknown to us. and our conclusions about the natal breeding grounds of these individuals are based on previously published data. As the breeding population of mallards around Ottenby is supposed to be small, and most of our individuals were caught during fall, according to banding recoveries and stable isotope analyses, originate from a corridor northwest of Ottenby [31], yet we do not know the individuals’ histories. That many ducks stayed at Lake Constance in spring might be indicative of their flexibility in choosing a place to breed, but could also be explained by so-called ‘homing attachment’ [35], to the release area. Homing attachment, or the likelihood to return to the native flyway has been shown in translocated North American ducks [35], and depends on the time spent in the release area, compared to the time spent in other places, because the tendency to migrate back to the original flyways and breeding grounds increased with age [35]. Although the hypothesis of two different migration strategies, with immediate correction in the first, and a later correction for translocation in the second group is viable, an alternative explanation is possible, namely that we caught and released birds from two distinct breeding populations. This explanation would not allow us to make conclusions about the navigational abilities of the birds, as we don’t know where the birds came from. However, these cases are not distinguishable from the data available, and since populations all over Eurasia resemble each other to a high extent, discrimination by genetic means would not be possible [36]. Overall, we observed a great amount of flexibility in mallards, in both their navigation strategies and their decisions to migrate in both winter and spring. Different responses to conditions and treatment could be observed both between, but also within groups, suggesting an individual, condition-dependent mechanism controlling the bird’s reactions to treatment and environment. The two strategies observable in spring migration is an interesting pattern, and it can be assumed that either birds have differing navigational ability, or were members of two groups with separate origins. Investigating navigation in ducks in closer detail might thus reveal intra-specific differences in navigation, and why some individuals are more likely to migrate than others.

## Materials and Methods

### Study Species and Study Sites

We used mallards, a dabbling duck with a wide breeding range, omnivorous food choice and flexibility with respect to habitat choice [29] In Europe, where the species naturally occurs, it is a partially migratory species. Temperate populations are often sedentary, whereas more northerly populations generally migrate in a south-westerly direction [37]. There is, however, a continuous intermixing between populations [36], as males can follow females from the common wintering sites to their breeding area [30]. Due to banding efforts, we know that mallards caught passing through southern Sweden in fall originate from breeding grounds in north-western Russia, the Baltic and southern Finland [31]. After a refueling period, they tend to continue migration in a south-westerly direction, heading towards northern Germany, Denmark and the Benelux states [31], [32], [38]. Lake Constance is a good-quality habitat for a diversity of wintering waterbirds and provides the transported ducks with similarly good conditions as the Ottenby nature reserve. Monthly waterbird censuses reveals approximately 18,000 mallards during late autumn (October) and 15,000 mallards in mid winter (January; data Ornithologische Arbeitsgemeinschaft Bodensee). Mallards ringed in southwest Germany show a wide spread of migration directions, but generally a southwest-northeast axis is visible from ringing recoveries (see Figure 1, data provided by the banding center Radolfzell, Germany).

### Animal Handling and Data Acquisition

All ducks were caught in a permanent baited duck trap at Ottenby that has been in use since the early 1960s. Details of trapping conditions are found in previously published articles [38]–[40]. Animals were sexed and aged according to differences in soft parts and plumage [41]. Seventy-six juvenile females with a bodyweight larger than 900 g were equipped with either 22 or 30 g solar-powered Argos/GPS platform transmitter terminals (Microwave Telemetry Inc., Columbia, MD, USA). In neither case did the tag-weight exceed 3.3% of the animals’ bodyweight (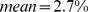). The tags were placed onto the back of the ducks and were fixed with Teflon band harnesses [42], the whole procedure took about ten minutes and did not exceed 20 minutes. The control ducks were released at the local capture site immediately after treatment (release site: N 56°13′16.13′′; E 16°26′33.88′′), while the test ducks were put into transport boxes and transported to Lake Constance with a private airplane. The complete transport took about eight hours via car and airplane (1 h transport to Kalmar, 1 h check-in time, 4 h flight, 2 h transport to the release site). In the first year, test ducks were released during the evening of arrival (11/30/2008). In the second year, however, they were released in the early morning of the following day (11/10/2009) to avoid releasing them in the dark after a late arrival. The release site was a shallow and food-rich place near Radolfzell (release site: N 47°44′19.21′′; E 088°59′5.17′′). We closely watched the ducks’ behavior during release, as translocation can significantly alter an animal’s stress response and reduce its survival [43]. All ducks appeared to be in good condition upon release. Some individuals could also be observed later and appeared to behave normally. Positional data of the ducks were acquired via ARGOS (www.argos-system.org) and uploaded into Movebank (www.movebank.org) for data management. The data used in this study are published in the Movebank Data Repository with DOI 10.5441/001/1.8dc0v84m [44]. Permission of capture and translocation of the ducks as well as their handling was permitted by Malmö-Lund University ethical committee for animal research (application number: M27-10).

### Tracking Results

Out of the 76 tags deployed over the two study years, GPS fixes were acquired for 60 of them, with a total number of 12’208 fixes. Tracking success was biased towards Radolfzell, with 35 working tags and 8’270 fixes (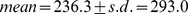 fixes per individual), and 25 working tags in Ottenby yielding a total of 3’938 fixes (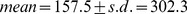 fixes per individual). Out of these 60 tracks available, only 34 tags had lasted long enough for further analysis of migratory behavior (at least 30 days of data collection after release), and hence all other tracks were removed from the analysis, except if a migratory event was evident. Additionally, three tags could be recovered after individuals were killed by hunters, and the recovery sites were included in the data. All further analyses were based on a total number of 40 animals, with 14 tracks from Ottenby and 26 tracks from the birds translocated to Lake Constance.

### Data Analysis

#### (a) Proportion of migratory ducks

As we were interested in whether the ducks would continue migration after release, we compared the proportion of ducks migrating during the winter after release between the two release sites. This was done by using a Fisher’s exact test for count data on the real numbers of winter migrating ducks for both years combined. As we expected the ducks to migrate back to their breeding grounds, we did the same test for the first spring following the experiment. Duck movement was rated as winter migration event when the distance between the first location after release and the last location in January were at least 50 km apart. For spring migration the condition for movement to be considered as migration was a minimum distance of 50 km between the mean location of March 1–15th and the last location of April. We based the estimate for minimum migration distances on knowledge about dispersal distances (natal dispersal: 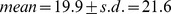 km) in mallards [45].

#### (b) Start and end point of migration and migration directions

For every duck classified as migratory, we determined the most likely start and end point of a migratory event visually. The last fix before traveling a considerable distance was considered as the beginning of migration. In most cases this is a sufficient approximation, as ducks usually started migrating during the night, where no GPS fixes were scheduled because of power requirements of the tracking tag. When the GPS tag recorded it’s first fix of the daily schedule, the ducks had already started migrating. The end of migration was either considered to be the first fix on the final wintering (for winter migration) or breeding site (for spring migration), or the last fix of the complete track, when the transmitter stopped sending data before the animal had reached its final destination. For the identification of the migration directions we extracted those parts of the tracks that were classified as migration, including stopovers, and calculated the mean bearing for the complete migration track weighted by distance. To identify the initial heading during each of the migratory events, we applied a change point approach. For this, we calculated the turning angle between every two bearings of the track and weighed them by the logarithm of the distance traveled along these bearings. Applying a sliding window to this vector of 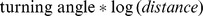, we fitted a normal distribution to the two fractions of the vector. The split for which Akaike’s information criterion (AIC) was minimal was considered the point at which there was a change in migration direction. We then calculated the mean initial heading of the animal for this first part of its migration, again weighted by the distance traveled. To achieve greater statistical power, we pooled the data of both years for the control as well as the translocated group and tested for uniform distribution of migration directions within and between groups using a Watson’s one-sample test for circular uniform distribution [46], [47] on the initial and complete migration directions for each season.

#### (c) Comparing weather conditions between sites

Surface temperature data for both release sites was obtained via Movebank for the complete study period. The data are derived from NOAA (reanalysis2) [48] and provide four estimates a day interpolated to the release sites from a 2.5 degree grid. We tested whether temperature conditions differed between sites using a one-sample t-test on the difference between weekly running means.

All analyses were done in R-2.15.0 [49].
